# Our Summer at Maplewood

**Published:** 1875-02

**Authors:** 


### OUR SUMMER AT MAPLEWOOD.

### BY COUSIN KATE.

### CHAPTER I. HOW IT ALL HAPPENED.

My cousin Emeline, after a two years sojourn in Europe with her only child, Alice, had returned, and was loitering in New York, undecided as to where they should spend the summer.

Abroad, Alice had devoted herself assiduously to French, German, and Music, while her mother had employed much of her time in writing a novel.

One day in early June, a fortnight after their arrival in the American metropolis, Emeline, looking up from the book she had been reading, exclaimed :

" 'O for a lodge in some vast wilderness !' Another week of this horible din and confusion will be the death of me. Day and night is one unceasing uproar. Kate, where can we hide ourselves for three months and enjoy uninterrupted rest, while I revise that book of mine ?"

I answered her by asking, " Would you mind going to Westfield ?"

"I feel just now as if I would not mind going to the ends of the earth, if necessary, in search of undisturbed quiet. But why go to Westfield ? "

Because my friend Jennie Douglas has a beautiful place there, which has been shut up for a year, and will so remain for a year or two longer. The place is left in care of the gardener ; but I have leave to go there when I will, and stay as long as I see fit—why not bury ourselves there ? We might bribe the gardener and his wife to keep our presence a secret as much as possible."

"The very thing," responded Emeline. "Let us start to-morrow. But where shall we get our rations while there, and who will cook them for us ? If we instal a retinue of servants in the house, we may as well abandon all thought of rest or comfort."

"I have a plan," I said, after a moment's reflection, " which will help me carry out a pet project of my own, and at the same time enable us to dispense with servants."

"My adorable Kate ! proceed, divulge, explain. I am all eagerness to know what pet project of yours can be subserved by our burying ourselves for three months in some out of the way place. I fail to see how that will do away with the evils of intemperance, or give the suffrage to women."

"Emeline, in my wandering to and fro upon the earth, I have been in a great many houses, and have eaten of the labors of a great many cooks. But the houses that deserved to be called Homes, wherein genuine comfort and cheerful happiness dwelt peacefully together—I could count them all on my ten fingers. In searching for the cause of this great lack, I cannot attribute it entirely to the tyranny of man, or the servitude of woman. I am forced to the conclusion that women fail to appeciate the importance of the Home. That in their just rebellion against lives of drudgery and toil, they fail to see how much of this drudgery is self-imposed, and what a grand work each woman might do in her circumscribed sphere if she could make the Home attractive and desirable above other places, to husband, sons, brothers. I have an idea that if the women who are wives and mothers would bring all their powers to bear in the right direction—making it their first object to see that the Home is comfortable and attractive—a place where the husband can rest, and the sons find pleasure—that these wives and mothers would constitute the most effective crusaders against the rumseller—that the labors of the women who go forth to pray with and convert the rumseller, would in comparison with their labors count as nothing."

"Cousin Kate, I protest against being forced to listen to your lecture on moral reform. Come to the point. What is your pet project?"

"Why this:—you see, Cousin Emeline, no Home can be what it ought to be, unless there is a good cook in the establishment. A great many of the sins of the world are traceable to bad food. A carnal stomach has often more to do with crime then a carnal heart. A popular writer says, 'you may make houses enchantingly beautiful, hang them with pictures, have them clean, airy and convenient—but if the stomach is fed with sour bread and burnt meats, it will raise such rebellion that the eyes will see no beauty anywhere.' Indeed I cannot conceive of a man being a Christian husband or father who is fed regularly upon half-baked bread, burnt or raw beef, and vile tea and coffee. Comfort with most of us is essential to happiness. Why, I have seen the peace of a whole family destroyed for the day by having muddy coffee for breakfast. Now, Emeline, I'm coming to the point. For years this matter has been a burden upon my mind, day and night. I have been longing to reach the ears and hearts of my countrywomen upon this subject. At last the longing has taken shape and form, and I see now in what way I can approach them. As so much of health and happiness, as well as enjoyment, is dependent upon the food we eat, and as no Home can deserve the name without a well-ordered table, I have concluded to write a cook-book."

"Write a cook-book! Well, why in the name of all that is sensible, didn't you say so? But I can't for the life of me see how your writing a cook-book will remedy the evils of which you complain, or enable us to dispense with servants at Westfield. We have a perfect library of cook-books already, and of making them, there appears to be no end. It requires an ordinary life-time almost, to even glance at the thousands and tens of thousands of recipes our numberless cook-books contain, many of them proved perfect, and handed down from generation to generation, along with the family china and silver. Don't do it, Kate."

"Cousin Emeline, you fail to comprehend my purpose. I'm not going to add another to the list of abominations miscalled cook-books, in which it is impossible to find a recipe whereby an unskilled or inexperienced housewife can make a loaf of bread equal to that made by our best New York bakers. In this one, which I pick up at random, I find only eleven recipes in any way touching bread—not one of them minute and careful in detail,—while I find in it sixty for cake, and fifty-eight for pies and puddings. From glancing over our popular cook-books, one would be apt to conclude the leading prayer of their writers was : 'Give us this day our daily pastry.' In *my* cook-book I will deal with the essential articles of food. I will dilate upon the charms of bread, meats, and vegetables, at their best; and omit those non-essential and indigestible messes and mixtures that have been heretofore thrust so prominently forward. Cook-book makers seem to take it for granted that every body knows how to make bread, broil a steak, cook a potato, etc., etc. Yet these are the very things that are almost universally spoiled, and rendered unfit to eat, by the average housekeeper. There are plenty of women—ten to one I might say truthfully—who can get up nice cake—but can't make good bread, or broil a beef steak or mutton chop properly. 'A fair Charlotte-russe is easier to gain than a good cup of coffee; and you shall find a sparkling jelly to your desert where you sighed in vain for so simple a luxury as a well cooked potato.' In this retreat of ours we will have undisturbed sway in the kitchen as well as in the parlor, and I will do the cooking in order to illustrate the perfection of my recipes and methods."

"And a sorry time we will have, I fear, in eating the results of your experiments," retorted Emeline, "but no matter; anything is preferable to this horrid New York life. Let us call Alice, and see if she consents to our plan."

Alice not only consented, but was greatly delighted at the prospect of this novel summer in the country ; and still more that she would have an opportunity of penetrating into the mysteries of the kitchen, or, as she expressed it, "of dabbling in dough."

So the question was speedily settled. And armed and equipped with chintz wrappers, linen aprons and rubber gloves, we arrived in due season at Maplewood, where, in the grand old kitchen, Alice and I were to be found morning and evening, with rare exceptions, all through that delightful summer, learning our lessons in the science of cookery.

